# The antihypertensive effect of remote ischemic conditioning in spontaneously hypertensive rats

**DOI:** 10.3389/fimmu.2022.1093262

**Published:** 2023-01-12

**Authors:** Xiaohua Li, Changhong Ren, Sijie Li, Wenbo Zhao, Peifu Wang, Xunming Ji

**Affiliations:** ^1^ Department of Neurology, Aerospace center Hospital, Beijing, China; ^2^ Beijing Institute of Brain Disorder, Capital Medical University, Beijing, China; ^3^ Beijing Key Laboratory of Hypoxic Conditioning Translational Medicine, Xuan Wu Hospital, Capital Medical University, Beijing, China

**Keywords:** hypertension, ischemic conditioning, inflammation, immune system, spontaneously hypertensive rats

## Abstract

**Purpose:**

Limb remote ischemic conditioning (LRIC) may be an effective method to control hypertension. This study investigated whether LRIC decreases blood pressure by regulating the hypertensive inflammatory response in spontaneously hypertensive rats (SHR).

**Method:**

The SHR and aged-matched Wistar rats with different ages were randomly assigned to the SHR group, SHR+LRIC group, Wistar group, and Wistar + LRIC group. LRIC was conducted by tightening a tourniquet around the upper thigh and releasing it for three cycles daily (10 mins x3 cycles). Blood pressure, the percentage of monocytes and T lymphocytes, and the concentration of pro-inflammatory cytokines in the blood were analyzed.

**Results:**

The blood pressure of SHR was significantly higher than that of age-matched Wistar rats. LRIC decreased blood pressure in SHR at different ages (4, 8, and 16 weeks old), but had no effect on the blood pressure in Wistar rats. Flow cytometry analysis showed that blood monocytes and CD8 T cells of SHR were higher than those of Wistar rats. LRIC significantly decreased the percentage of monocytes and CD8 T cells in SHR. Consistent with the changes of immune cells, the levels of plasma IL-6 and TNF-α in SHR were also higher. And LRIC attenuated the plasma IL-6 and TNF-α levels in SHR.

**Conclusion:**

LRIC may decreased the blood pressure via modulation of the inflammatory response in SHR.

## Introduction

Hypertension affects approximately one third of the world’s adult population and is a major cause of premature death. It is an independent risk factor for numerous diseases, including coronary artery disease (CAD), stroke, chronic kidney disease, and peripheral arterial disease. High blood pressure is the most significant contributor to global deaths, disability-adjusted life-years (DALYs) (1, 2). The global prevalence of hypertension is still on the rise. The number of people aged 30-79 years with hypertension doubled from 1990 to 2019 (3). Hypertension is associated with a substantial financial burden. The global financial burden of high blood pressure in 2001 was estimated to be around 370 billion US dollars or about 10% of the world’s overall healthcare expenditure (4). Therefore, novel, cost-effective interventions for hypertension should be explored.

Limb remote ischemic conditioning (LRIC) is an easy-to-use and noninvasive strategy in which one or more cycles of brief limb ischemia followed by reperfusion confer protection against subsequent, more severe ischemia in distant organs (5, 6). Some studies (7, 8) have indicated that long-term LRIC may have the potential antihypertensive effect. Our previous study also revealed that chronic LRIC decreased blood pressure in spontaneously hypertensive rat (SHR) and prehypertensive patients (9). Therefore, LRIC may be a potential way to control hypertension. However, it is not clear whether LRIC has antihypertensive effect on progressive hypertension and its possible mechanism. LRIC may be a new method worthy of further exploration.

Inflammation is a pivotal cause of hypertension and hypertensive end-organ damage. Both the innate and adaptive immune systems play an essential role in the pathogenesis of hypertension (10, 11). Macrophages and T lymphocytes infiltrate vasculature and kidney, producing several pro-inflammatory cytokines, including TNF-α, IL-17, IFN-γ, and IL-6. Cytokines in blood vessels and kidneys cause vasodilation dysfunction and urinary sodium excretion disorder, leading to hypertension (12–15). Modulating the immune response can reduce the blood pressure elevation and hypertensive end-organ damage (16–18). Decreasing systemic or tissue inflammation is an important protective mechanism of LRIC (19). LRIC could inhibit the increase of cytokines (TNF-α, IL-6, and IL-1) in the LPS-injected mice (20). LRIC also reduced circulating CD3+/CD8+ T cells and NK cells following stroke (21). Genes encoding key proteins involved in cytokine synthesis, innate immunity signaling pathways, and apoptosis were all suppressed by LRIC (22). These studies support a close link between inflammation and LRIC-mediated protection. So, we speculate that regulating the immune system may be the antihypertensive mechanism mediated by LRIC. We try to testify whether LRIC decreases blood pressure by modulating the hypertensive inflammatory response.

## Methods

### Animals

All animal experiments were approved by the Animal Care and Use Committee of Xuanwu Hospital, Capital Medical University, China, and conducted according to the National Institutes of Health guidelines. Adult male spontaneously hypertensive rats (SHR) and Wistar rats, aged 4, 8, and 16 weeks were purchased from Vital River Laboratories, Beijing, China, and maintained on a 12-hour light/dark cycle with unlimited access to food and water. The SHR and age-matched Wistar rats were randomly assigned to the LRIC group (SHR LRIC and Wistar LRIC) and control group (SHR CON and Wistar CON).

### Limb remote ischemic conditioning

LRIC was conducted as previously described (23). LRIC was conducted on SHR and Wistar rats of different ages (4, 8, and 16 weeks old) every day until no observed difference in blood pressure among various groups. Before each LRIC treatment, the rats both in control and LRIC group were anesthetized with sodium pentobarbital (30 mg/kg) intraperitoneally. Then, LRIC was conducted in treatment group by tightening a tourniquet around the bilateral upper thigh to prevent blood flow and release it for three cycles every day, with each occlusion or release phase lasting 10 minute. The rats in the control groups only undergo anesthesia as in the LRIC group.

### Blood pressure measurement

The systolic blood pressure (SBP), diastolic blood pressure (DBP), mean arterial pressure (MAP) and heart rate (HR) were measured weekly in the conscious rats by the noninvasive tail-cuff system (SoftronBP-2010A; Softron, Tokyo, Japan), according to the manufacturer’s instructions. Once a week, the blood pressure of each rat was measured three times consecutively, and the average value was taken as the final blood pressure.

### Flow cytometry

Immunocytes in the blood were analyzed by flow cytometry following standard protocols. Briefly, 100μL of whole anticoagulant blood was incubated with mixed antibodies (Rat T Lymphocyte Cocktail CD3 APC/CD8a FITC/CD4 PE, BD, 20ul/T; Anti-Rat CD172a (SIRP alpha) APC, eBioscience, 1.25ul/T; PE anti-rat CD43 Antibody, eBioscience, 0.3ul/T) at room temperature (RT) for 20 min in the dark, then the blood samples were lysed in lysis buffer at RT for 10 min, followed by a wash with PBS. Then, the samples were assayed according to the manufacturer’s instructions with the FACS Fortessa flow cytometer (BD Bioscience, San Jose, CA, USA.)

### Measurement of circulating cytokines

Blood was collected into a vacuum tube coated with EDTA from the left ventricle and then centrifuged at a speed of 3000 rpm for 20min at 4°C. Plasma was obtained and stored at -80°C. Cytokines, including IL-6, IL-17, TNF-α, and INF-γ in plasma, were quantified using the Rat Cytokine/Chemokine Magnetic Bead Panel (Millipore, USA) according to the manufacturer’s instructions.

### Serum extraction and caudal vein injection

After two weeks of LRIC and placebo treatment to 8-week-old SHR rats, the blood was collected into a vacuum tube without anticoagulant from the left ventricle and then centrifuged at a speed of 3000 rpm for 20min after at 4°C. The supernatant was retained and stored at -80 °C for caudal vein injection. The serum (500 μ L) from SHR LRIC or SHR CON rats was injected to 8-week-old SHR through the tail vein every day for seven days.

### Statistical analysis

Statistical analysis was performed with SPSS16.0. Parameters were expressed as a mean ± standard deviation (SD). Comparisons of blood pressure across time points were analyzed by two-way repeated-measures ANOVA. Other results were analyzed with an independent sample *t-Test *(for two groups) and one-way ANOVA followed by Fisher’s LSD *post hoc* test (for multiple groups). Statistical significance is shown as *p < 0.05, ** *p *< 0.01, or *** *p* < 0.001.

## Results

### LRIC prevented the blood pressure increase in 4 weeks old SHR

**Figure 1:**
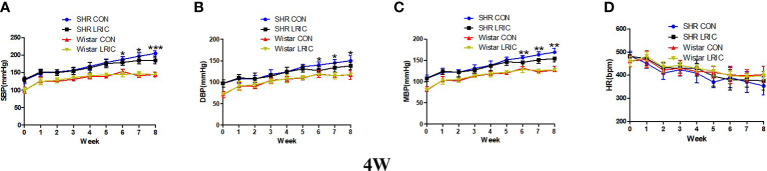
LRIC prevented the blood pressure increase in 4 weeks old SHR. **(A)** Systolic blood pressure (SBP) in 4 weeks** **old SHR and Wistar rats. **(B)** Diastolic blood pressure (DBP) in 4 weeks** **old SHR and Wistar rats. **(C)** Mean blood pressure (MBP) in 4 weeks** **old SHR and Wistar rats. **(D)** Heart rate (HR) in 4 weeks old SHR and Wistar rats. Values are mean ± SD. Blue line: SHR CON group, N=9; Black line: SHR LRIC group, N=9; Red line: Wistar CON group, N=7; Yellow line: Wistar LRIC group, N=7. (*p<0.05, **p<0.01, ***p<0.001, one-way ANOVA with Fisher’s LSD multiple comparisons test.).

The blood pressure of SHR begins to increase as early as the fourth week after birth. As shown in **Figure 1**, the SBP (**Figure 1A**, 130 ± 7.5 vs. 99 ± 7.6mmHg, *p* < 0.001), DBP (**Figure 1B**, 98 ± 8.1vs. 73 ± 2.0mmHg, *p* < 0.001), and MBP (**Figure 1C**, 108 ± 7.6 vs. 81 ± 3.8mmHg, *p* < 0.001) of 4 weeks old SHR was significantly higher than that of age-matched Wistar rats. For Wistar rats, LRIC had no pronounced effect on blood pressure and HR (**Figure 1**, *p* > 0.05). For SHR, LRIC significantly prevented the SBP (**Figure 1A**, 179 ± 11.8 vs. 188 ± 10.7mmHg, *p* < 0.05), DBP (**Figure 1B**, 127 ± 12.6 vs. 140 ± 7.1mmHg, *p* < 0.05) and MBP (**Figure 1C**, 145 ± 9.9 vs. 156 ± 4.8mmHg, *p* < 0.01) increase after six weeks of treatment but did not influence the HR (**Figure 1D**, *p >*0.05). However, LRIC could not decrease blood pressure to a normal level compared with Wistar rats.

### LRIC inhibited the blood pressure increase in 8 weeks old SHR

**Figure 2:**
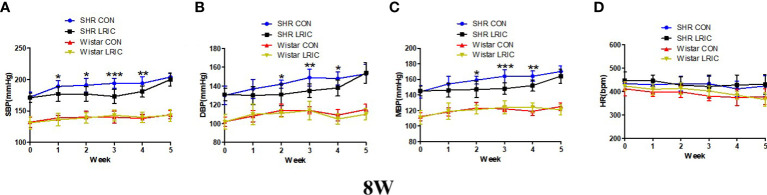
LRIC inhibited the blood pressure increase in 8 weeks old SHR. **(A)** Systolic blood pressure (SBP) in 8 weeks** **old SHR and Wistar rats. **(B)** Diastolic blood pressure (DBP) in 8 weeks** **old SHR and Wistar rats **(C)** Mean blood pressure (MBP) in 4 weeks** **old SHR and Wistar rats. **(D)** Heart rate (HR) in 8 weeks old SHR and Wistar rats. Values are mean ± SD. Blue line: SHR CON group, N=9; Black line: SHR LRIC group, N=9; Red line: Wistar CON group, N=7; Yellow line: Wistar LRIC group, N=7. (*p<0.05, **p<0.01, ***p<0.001, one-way ANOVA with Fisher’s LSD multiple comparisons test.).

The blood pressure in SHR increases rapidly between 8-12 weeks. We implement LRIC in 8 weeks old SHR and Wistar rats to determine whether LRIC plays a similar inhibiting effect as in 4 weeks old SHR. Results showed that LRIC training did not change the blood pressure in the Wistar rats and had no effect on HR in both SHR and Wistar rats (**Figure 2**, *p* > 0.05). LRIC significantly inhibits blood pressure increase in 8 weeks old SHR after one week of intervention (**Figure 2A**, SBP: 177 ± 9.4 vs. 189 ± 10.0mmHg, *p* < 0.05). This effect lasted for four weeks. The maximum difference in blood pressure was observed after three weeks of LRIC training (**Figure 2A**, SBP: 173 ± 8.0 vs. 194 ± 9.0mmHg, *p* < 0.001; **Figure 2B**, DBP: 135 ± 9.1 vs. 149 ± 7.1mmHg, *p* < 0.01; **Figure 2C**, MBP: 148 ± 8.4 vs. 164 ± 7.3mmHg, *p* < 0.001). LRIC induced a significant mean drop of MBP by 16mmHg. However, the blood pressure of SHR was still significantly higher than that of Wistar rats. And the blood pressure difference between the group of SHR LRIC and SHR CON disappeared in the fifth week of treatment.

### LRIC decreased the developed hypertension in 16 weeks old SHR

**Figure 3:**
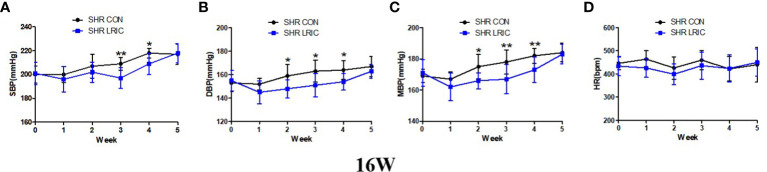
LRIC decreased the developed hypertension in 16 weeks old SHR. **(A)** Systolic blood pressure (SBP) in 16 weeks** **old SHR. **(B)** Diastolic blood pressure (DBP) in 8 weeks** **old SHR. **(C)** Mean blood pressure (MBP) in 16 weeks** **old SHR. **(D)** Heart rate (HR) in 16 weeks old SHR and Wistar rats. Values are mean ± SD. Blue line: SHR CON group, N=9; Black line: SHR LRIC group, N=9; (*p<0.05, **p<0.01, two sample independent sample t-Test.).

The sustained hypertension was developed at 16 weeks in SHR. So LRIC training was given to 16 weeks old SHR to determine whether LRIC lower established hypertension. The results revealed that the HR (**Figure 3D**) of 16 week old rats was also not affected by LRIC treatment, After two weeks of LRIC, the DBP (**Figure 3B**, 163 ± 9.4 vs. 151 ± 10.0mmHg, *p* < 0.05) and MBP (**Figure 3C**, 178 ± 7.8 vs. 167 ± 9.5mmHg, *p* < 0.01) in the LRIC group was significantly decreased compared with the control group. After three weeks of LRIC treatment, the SBP was lower in the LRIC group than in the control group (**Figure 3A**, SBP: 209 ± 5.3 vs. 197 ± 8.6mmHg, *p* < 0.01). This difference disappeared in the fifth week of treatment.

### LRIC attenuated the circulating monocytes, and CD8 T cells increase in SHR

**Figure 4:**
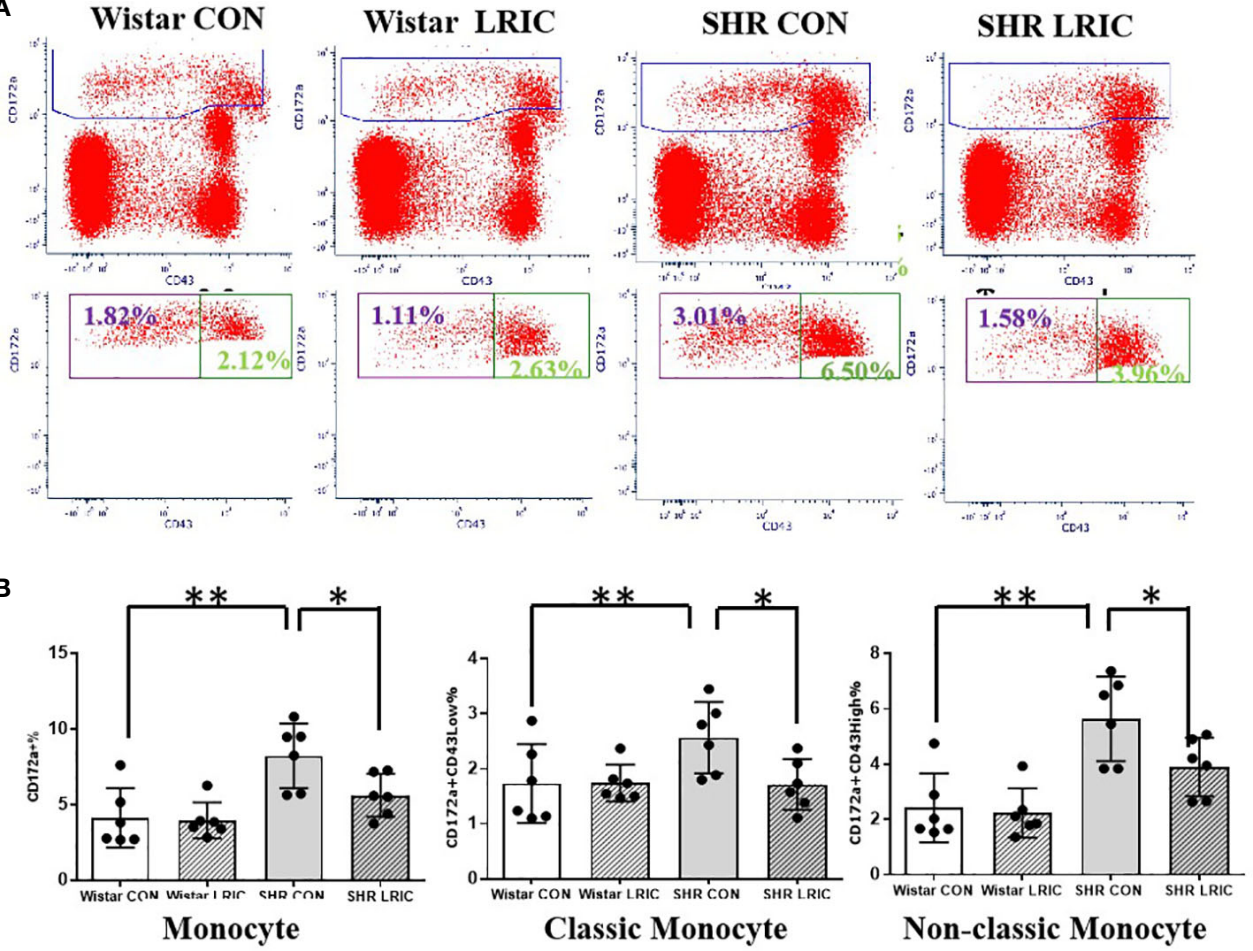
LRIC attenuated the circulating monocytes increase in SHR. **(A)** Representative flow cytometry graphs of monocytes between different groups. **(B)** Statistical analysis of monocyte changes between different groups. Values are mean ± SD. N=6; (*p<0.05, **p<0.01, one-way ANOVA with Fisher’s LSD multiple comparisons test.).

We detected the level of monocytes and T lymphocytes in circulation after two weeks of LRIC and placebo treatment to 8 weeks old SHR and Wistar rats. As shown in **Figure 4**, the monocyte percentage in SHR was remarkably higher than in Wistar rats (**Figure 4**, 8.22 ± 0.87% vs. 4.12 ± 0.80%, *p* < 0.01). LRIC treatment significantly decreased the monocyte percentage in SHR (**Figure 4**, 5.60 ± 0.58% vs. 8.22 ± 0.87%, *p* < 0.05), and this distribution was consistent in classic and non-classic monocytes. LRIC did not change the monocytes counts in Wistar rats (**Figure 4**, 3.90 ± 0.48% vs. 4.1 ± 0.80%, *p* > 0.05).

**Figure 5:**
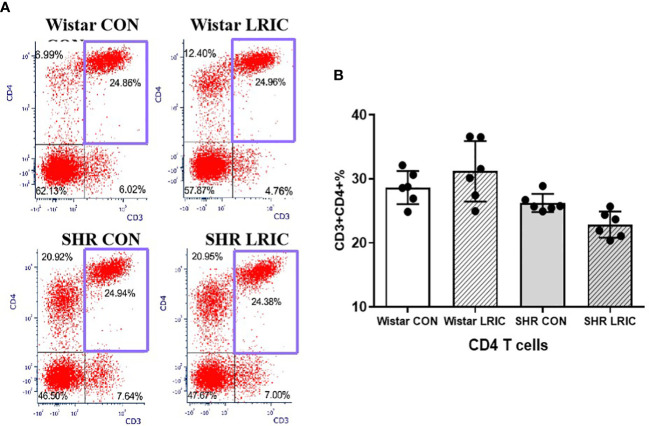
LRIC did not influence circulating CD4 T cells. **(A)** Representative flow cytometry graphs of CD4 T cells in different groups. **(B)** Statistical analysis of CD4 T cells changes between different groups. Values are mean ± SD. N=6. (one-way ANOVA with Fisher’s LSD multiple comparisons test.).

We identified CD3+ as T cell, CD3+CD4+ as Th cell, and CD3+CD8+ as Tc cell. There was no difference in the CD4 T cells between the SHR and the Wistar group (**Figure 5**, 28.65 ± 1.05% vs. 26.2 ± 1.42%, *p* > 0.05). LRIC did not influence the CD4 T cells percentage both in SHR (**Figure 5**, 22.8 ± 1.05% vs. 26.2 ± 1.42%, *p* > 0.05) and Wistar rats (**Figure 5**, 28.65 ± 1.05% vs. 31.2 ± 1.93%, *p* > 0.05).

**Figure 6:**
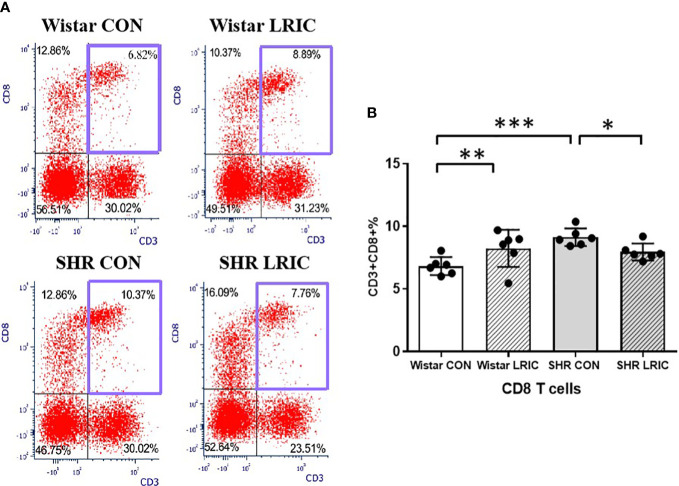
LRIC attenuated the circulating CD8 T cells increase in SHR. **(A)** Representative flow cytometry graphs of CD8 T cells in different groups. **(B)** Statistical analysis of CD8 T cells changes between different groups. Values are mean ± SD. N=6. (*p<0.05, **p<0.01, ***p<0.001, one-way ANOVA with Fisher’s LSD multiple comparisons test.).

The percentage of CD8 cells in SHR CON was higher than that in the Wistar CON (**Figure 6**, 6.82 ± 0.71% vs. 9.12 ± 0.70%, *p* < 0.01). LRIC significantly decreased the percentage of CD8 T cells in SHR (**Figure 6**, 7.95 ± 0.67% vs. 9.12 ± 0.70%, *p* < 0.05). However, LRIC increased the CD8 cells percentage in Wistar rats (**Figure 6**, 6.82 ± 0.71% vs. 8.24 ± 1.48%, *p* < 0.01).

### LRIC decreased the plasma TNF-α and IL-6 levels in SHR

**Figure 7:**
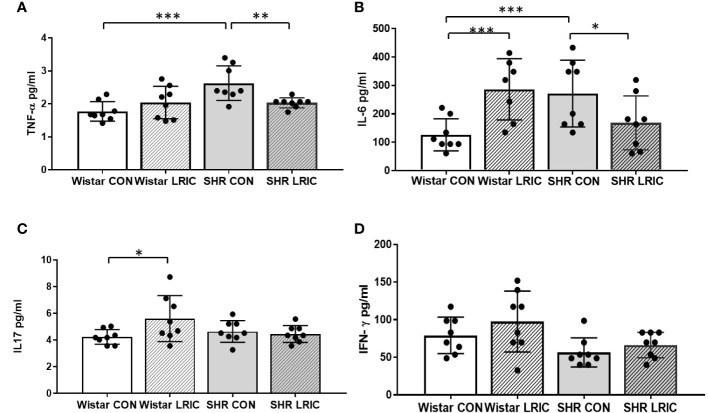
LRIC decreased the plasma TNF-α and IL-6 levels in SHR. **(A)** The plasma TNF-α level in different group. **(B)** The plasma IL-6 level in different groups. **(C)** The plasma IL-17 level in different groups. **(D)** The plasma IFN-γ level in different groups. Values are mean ± SD. N=8/Group. (*p<0.05, **p<0.01, ***p<0.001, one-way ANOVA with Fisher’s LSD multiple comparisons test.).

We measured the level of plasma inflammatory cytokines after two weeks of LRIC and placebo treatment to 8-week-old SHR and Wistar rats. The level of plasma TNF-α (**Figure 7A**, 1.77 ± 0.29 vs. 2.63 ± 0.52 pg/ml, *p* < 0.001) and IL-6 (**Figure 7B**, 126 ± 56.3 vs. 271 ± 117.5 pg/ml, *p* < 0.001) in SHR were higher than those in Wistar rats. LRIC both decreased the plasma TNF-α (**Figure 7A**, 2.03 ± 0.15 vs. 2.63 ± 0.52 pg/ml, *p* < 0.01) and IL-6 levels (**Figure 7B**, 168 ± 94.9 vs. 271 ± 117.5pg/ml, *p* < 0.05). However, LRIC increased the plasma IL-6 level (**Figure 7B**, 286± 107.9 vs. 126 ± 56.3 pg/ml, *p* < 0.01) in Wistar rats. There was no difference in plasma IL-17 (**Figure 7C**, 4.64 ± 0.80 vs. 4.23 ± 0.54 pg/ml, *p* > 0.05) and IFN-γ levels (**Figure 7D**, 56 ± 19.3 vs. 79 ± 24.4, pg/ml, *p* > 0.05) in SHR and Wistar rats. LRIC did not change the level of IL-17 (**Figure 7C**, 4.64 ± 0.80 vs. 4.45 ± 0.63 pg/ml, *p* > 0.05) and IFN-γ (**Figure 7D**, 66 ± 16.8 vs. 56 ± 19.3 pg/ml, *p* > 0.05) in SHR. However, LRIC increased the plasma IL-17 level (**Figure 7C**, 5.6 ± 1.7 vs. 4.23 ± 0.54pg/ml, *p* < 0.05) in Wistar rats.

### The serum from LRIC treated SHR decreased blood pressure in 8 weeks old SHR

**Figure 8:**
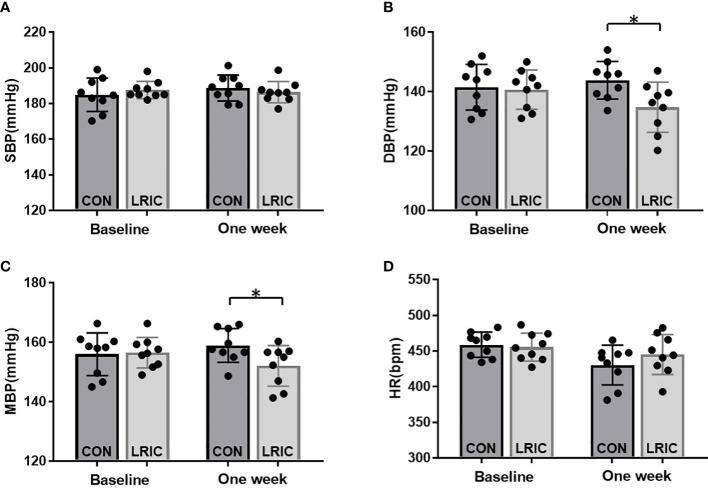
The serum from LRIC treated SHR decreased blood pressure in 8 weeks old SHR. **(A)** The serum from LRIC treated SHR had no effect on SBP in 8 weeks old SHR. **(B)** The serum from LRIC treated SHR significantly decreased the DBP in 8 weeks old SHR. **(C)** The serum from LRIC treated SHR significantly decreased the MBP in 8 weeks old SHR. Values are mean ± SD. N=9. (*p<0.05, two sample independent sample t-Test.).

Various studies revealed that LRIC exerts protective effects by humoral pathways (22). Therefore, in this study, we tried to transfer the serum collected from LRIC treated SHR to 8 weeks old SHR and then observed a blood pressure change. The results showed that the serum from LRIC treated SHR significantly decreased the level of DBP (**Figure 8B**, 144 ± 6.3 vs. 134 ± 8.4mmHg, *p* < 0.05) and MBP (**Figure 8C**, 159± 5.6vs. 152 ± 6.8mmHg, *p* < 0.05) in 8 weeks old SHR. However, the serum from LRIC treated SHR did not alter the SBP (**Figure 8A**, 186 ± 5.9vs 188 ± 7.2mmHg, *p* > 0.05) and HR (**Figure 8D**, *P*>0.05) in SHR.

## Discussion

In this study, we demonstrated that long-term LRIC delayed the blood pressure increase in young SHR and decreased the blood pressure in 16 weeks old SHR with established hypertension. LRIC approximately lowers blood pressure by 5-21mmHg in SHR of different ages. Moreover, this antihypertensive effect lasted for about four weeks. Furthermore, LRIC reduced the monocytes and CD8 T cells counts and the TNF-α and IL-6 levels in blood.

Both innate and adaptive immune systems contribute to hypertension (10, 11). Monocytes/macrophages are primary effectors of innate immunity (24). Monocyte aggregation in the vascular system and kidneys of hypertensive animals and humans has been widely observed (25). Our present data indicated that the monocyte counts in circulation increased in SHR, and LRIC alleviated this increase. Convincing evidence has suggested that T lymphocytes, categorized into CD4 T lymphocytes (Th cells) and CD8 T lymphocytes (Tc cells), contribute to blood pressure elevation (11, 26, 27). However, which T cell subtype is involved in the regulation of hypertension is still controversial. Some studies (26, 28) revealed that mice lacking CD8+ T cells, not CD4+ T cells blunt Ang II-induced BP elevation, and transfer of CD8 but not CD4+ T cells into Rag1-deficient animals re-establish the hypertensive response to chronic angiotensin II infusion, suggesting that CD8 T cells may be necessary for hypertension. Our results also displayed that the circulating CD8+ T cells increased in SHR, and LRIC significantly attenuated the CD8+ T cells increase in SHR.

A previous study (22) showed that LRIC down-regulated the expression of pro-inflammatory cytokines in normal subjects, but in our study LRIC increased the number of CD8+T cells and the levels of IL-17 and IL-6 in normal Wistar rats. In our study, the inflation pressure of LRIC on different groups was the same. Therefore, the Wistar rats received relatively higher pressure. We speculated that excessive pressure may leads to an immune response in Wistar rats. Therefore, the optimal inflation pressure of LRIC and the effect of LRIC on healthy subject still need to be confirmed in future research.

Inflammatory cytokines like IL-6, TNF-α, IFN-γ, and IL-17 released by immune cells significantly increase in patients with hypertension (29), and the levels of plasma IL-6 and TNF- α was positively correlated with blood pressure and end-organ damage (30). Consistent with previous results, our data also revealed that the plasma IL-6 and TNF-α levels were elevated in SHR. Furthermore, LRIC treatment decreased the upregulated IL-6 and TNF-α levels in SHR. However, we did not observe a difference in the level of IFN-γ and IL-17.

The humoral mechanism is an essential pathway for RIC-induced protection. Such as the transfer of blood from an LRIC rabbit reduced myocardial infarction (31). However, despite various of circulating factors that have been proposed as contributors, the exact nature of the humoral factor has not been identified. One study demonstrated that transfusion of the platelet-derived microparticles prepared from healthy rats subjected to hindlimb RIPC could protect the recipient mice against ischemic brain injury (32). These microparticles are known to carry a rich set of cytokines, chemokines, enzymes, and signaling protein (33). Our results also indicated that the transfusion of the serum after LRIC treatment decreased the blood pressure in SHR. So, whether peripheral pro-inflammatory factors are the essence of humoral factors induced by LRIC needs further verification.

Our results suggest that LRIC may provide antihypertensive effect by mobilizing the peripheral immune system. How LRIC induced the alterations in peripheral immune system remains elusive. Previous study showed that microglia may bridge the central and peripheral inflammation *via* regulating the sympathetic nerve activity in hypertension. Increased microglial activation1 and splenic cytokine gene expression was observed after Ang II administration (34). And the ablation of microglia reduces RAS-induced inflammation and prevents hypertension (35). Microglia are crucial effectors of ischemic conditioning mediated protection. Post ischemic conditioning promotes microglia/macrophage polarization into potentially beneficial phenotype after stroke (36). So, we speculated that glia, especially microglia may be the trigger of peripheral immune response induced by LRIC which need to be clarified. There are still questions remain to be answered. First, it is necessary to use specific cell adoptive transfer and depletion to further evaluate the role of the immune cells in LRIC induced antihypertensive effect. Second, other immune cells and cytokines, including NKT, T-reg, IL-1, and IL-10, should be detect. Third, the reason of disappearance of antihypertensive effect induced by LRIC after one month needs to be clarified.

In conclusion, our study indicated that LRIC exerts an antihypertensive effect by modulating peripheral inflammatory response in SHR. Despite considerable advances in pharmacological treatments, more individuals are resistant hypertension among the treated hypertensive population (37). LRIC is a simple, non-invasive, and cost-effective method. Thus, our work provides a possible novel intervention for the prevention and treatment of hypertension.

## Data availability statement

The raw data supporting the conclusions of this article will be made available by the authors, without undue reservation.

## Ethics statement

The animal study was reviewed and approved by Animal Care and Use Committee of Xuanwu Hospital, Capital Medical University.

## Author contributions

JXM and CR were responsible for the study conception and design. WZ and PW contributed to data extraction and assessment of study quality. XL, and SL contributed analysis and interpretation of data, and draft manuscript. All authors contributed to the article and approved the submitted version.
